# Dendritic Cell Migration Is Tuned by Mechanical Stiffness of the Confining Space

**DOI:** 10.3390/cells10123362

**Published:** 2021-11-30

**Authors:** Yongjun Choi, Jae-Eun Kwon, Yoon-Kyoung Cho

**Affiliations:** 1 Department of Biomedical Engineering, Ulsan National Institute of Science and Technology (UNIST), Ulsan 44919, Korea; yzest@unist.ac.kr; 2 Center for Soft and Living Matter, Institute for Basic Science (IBS), Ulsan 44919, Korea; 3 Department of Material Science and Engineering, Ulsan National Institute of Science and Technology (UNIST), Ulsan 44919, Korea; kje001128@unist.ac.kr

**Keywords:** dendritic cell, cell migration, stiffness, elastic modulus, confinement, hydrogel

## Abstract

The coordination of cell migration of immune cells is a critical aspect of the immune response to pathogens. Dendritic cells (DCs), the sentinels of the immune system, are exposed to complex tissue microenvironments with a wide range of stiffnesses. Recent studies have revealed the importance of mechanical cues in immune cell trafficking in confined 3D environments. However, the mechanism by which stiffness modulates the intrinsic motility of immature DCs remains poorly understood. Here, immature DCs were found to navigate confined spaces in a rapid and persistent manner, surveying a wide range when covered with compliant gels mimicking soft tissues. However, the speed and persistence time of random motility were both decreased by confinement in gels with higher stiffness, mimicking skin or diseased, fibrotic tissue. The impact of stiffness of surrounding tissue is crucial because most in vitro studies to date have been based on cellular locomotion when confined by microfabricated polydimethylsiloxane structures. Our study provides evidence for a role for environmental mechanical stiffness in the surveillance strategy of immature DCs in tissues.

## 1. Introduction

Dendritic cells (DCs) patrol the tissue microenvironment, acting as immune sentinels to search for and phagocytose pathogens. Since this surveillance by immature DCs is the basis of the innate immune response, it is crucial to understand the physical and biochemical factors that influence it. Previous studies have demonstrated that DC motility is modulated by external factors including cytokines [1,2], pathogen components [3,4,5], and ATP released from dead cells [6]. Furthermore, recent studies suggest that mechanical and geometrical cues, including confinement, rigidity, topology, and porosity of the microenvironment, modulate DC motility [7,8] and function [9]. 

Geometric confinement has been recognized as a critical parameter for controlling the characteristic ability of fast amoeboid migration of DCs in vivo [8]. The directional motility of DCs confined in polydimethylsiloxane (PDMS) microchannels was found to be controlled by chemotaxis [3,10,11] and barotaxis [12], guided by the concentration and pressure gradients generated in the narrow cavity. Haptotaxis—directional motility guided by an adhesion molecular gradient—was suggested by a study using a microfabricated PDMS structure with patterning of adhesive molecules [13,14]. A complex tissue microenvironment, in which cells encounter multiple pores with different geometric resistances, was mimicked using bifurcation microchannels with pores of 2 and 5 µm in width, which showed that DCs predominantly migrated through larger pores [15]. 

The intrinsic motility of DCs in the absence of directional cues has also been intensively studied to understand their space exploration strategies [16,17,18]. For example, by quantifying the speed fluctuation of immature DCs under confinement within microchannels, it has been shown that migration and antigen uptake are antagonistic processes [18]. Intermittent migration has also been reported, alternating diffusive and persistent motility for an efficient search strategy [16,17]. A topological difference in microstructure induces adaptive DC motility changes when coupling the intracellular force of the actin cytoskeleton to the environment [19]. Nevertheless, most experiments have been conducted using hard PDMS microstructures, limiting our understanding of cellular locomotion in soft tissues.

Tissues in the human body exhibit a broad range of stiffness, spanning Young’s elastic moduli of 1 kPa (e.g., brain) to 1 GPa (e.g., bone) [20]. The elastic modulus of most soft tissues is under 5 kPa, but several tissues, such as skin, lymph node, and spleen, have Young’s moduli over 10 kPa. In addition, multiple diseases including fibrosis, calcification, and cancer can disrupt homeostasis, increasing elastic moduli [21]. Despite this wide range of stiffness, DCs are found in almost all tissues, both healthy and diseased [22]. A recent study demonstrated that environmental stiffness modulates DC metabolism, phenotype, and inflammatory function [9]. These data suggest that DC phenotypes and motilities are altered as a function of the mechanical stiffness of their environment. 

While mature DCs have been the subject of most such studies, little is known about how external mechanical loads affect the intrinsic motility of immature DCs. Previous studies using stiffness-controlled substrates, such as polyacrylamide gel or PDMS micro-post arrays, only addressed mature DC motility, showing that motility was insensitive to substrate stiffness in the range tested (e.g., 2, 12, 50 kPa Polyacrylamide gel or 1.49, 17.21, 1218.44 kPa micro-post array) [23,24]. In this study, we reasoned that the effects of mechanical loads on DC migration must be evaluated in confined microenvironments and not on 2D substrates and investigated the motility of immature DCs under confinement.

Recently, we developed a “gel confiner”, which enables facile handling of agarose gel ceilings for more robust under-agarose assays [25]. We have used the gel confinement approach to tune the mechanical load imposed on immature DCs by changing the agarose concentrations. Instead of cells situated in open confined spaces in microfabricated PDMS structures, our DCs were fully confined by soft agarose gels with different stiffnesses and forced to adapt their cellular morphology upon mechanical interaction with the environment. This strategy potentially better mimics the in vivo interface between cells and complex tissues with different degrees of stiffness. We investigated how the mechanical load of the surrounding environment influences the cellular morphology and intrinsic motility patterns of immature DCs.

## 2. Materials and Methods

### 2.1. Preparation of Bone-Marrow-Derived DCs (BMDCs)

BMDCs were prepared as previously reported [10,25,26]. More details are presented in the Electronic Supplementary Information (ESI). Flow cytometry was used to measure the expression of CD11c and MHC class II (I-A/I-E) (Figure S1). For clear verification, immature DCs and lipopolysaccharide (LPS) (100ng/mL, LPS-EB Ultrapure, Invivogen, Hong Kong, China) treated DCs were investigated together to assess the upregulation of MHC class II.

### 2.2. Gel Confiner for Under-Agarose Assay

The fabrication of the gel confiner is described in the ESI and in a previous study [25]. Briefly, a custom designed PDMS structure was prepared as a frame to hold liquid agarose. After curing, the agarose gel became integrated with the PDMS structure without chemical bonding, so the gel confiner could be handled using the PDMS rim without touching the agarose. Approximately 800 cells were seeded on coverslips (10 mm diameter) coated with bovine fibronectin serum (FN; 20 μg/mL; Sigma, Burlington, MA, USA; cell density = 1.0 × 10^−4^ cells/μm^2^) and incubated for 20 min in a cell-culture incubator. The cell suspension was carefully washed three times with fresh complete medium (RPMI 1640) supplemented with 5% (*v*/*v*) fetal bovine serum (FBS), 1% (*v*/*v*) antibiotic–antimycotic solution, 1% (*v*/*v*) HEPES buffer, and 0.1% (*v*/*v*) 2-mercaptoethanol (all purchased from Gibco, Amarillo, TX, USA) and assembled with a gel confiner.

### 2.3. Measurement of Young’s Moduli

Agarose gel blocks were prepared at concentrations of 0.5%, 1%, or 1.5% (*w*/*v*). Mechanical properties of the gel were measured using a rheometer (MCR502 WESP; Anton Paar, Austria) (Figure S2). The thickness of the gel was set to approximately 1.0 mm. Shear moduli (G, Pa) were calculated using the relationship between shear stress and shear strain before gel disruption, and Young’s moduli (E, kPa) were calculated using the following equation: E = 2(1 + v)G, with the Poisson ratio (v) of agarose set as 0.5 [27].

### 2.4. Live-Cell Imaging

DCs were imaged using an inverted microscope (Eclipse Ti-E, Nikon, Melville, NY, USA) configured with a 10× *g* dry objective lens and CMOS camera (Flash 4.0; Hamamatsu, Hamamatsu, Japan). Cell trajectories were recorded after 1 h, capturing bright-field images every 1 min for 12 h in a custom incubator system (Chamlide HK; Live Cell Instrument, Seoul, Korea) maintained at 37 °C with 5% CO_2_. 

### 2.5. DC Motility

Motility was measured using Imaris (Bitplane, Zürich, Switzerland) and a custom MATLAB (MathWorks, Natick, MA, USA) code that was modified from a previous report [28]. Mean track speed was calculated by dividing track length by track duration. Instantaneous speed was measured by dividing the length between two consecutive frames by the corresponding time difference. The standard deviation of the instantaneous track speed was divided by mean track speed to calculate speed fluctuation. The maximum distance between the origin of the track and the farthest position was defined as the travel range. The mean square displacement (MSD) of the cell trajectory was calculated as anomalous diffusion, and power-law fit was calculated according to the formula MSD (τ) = 4Dτ^α^, where D is the diffusion coefficient (μm^2^/min), τ is the time lag (min), and α is the scaling exponent [29]. The random Brownian motion was described as α = 1. Other motions were described as subdiffusive (α < 1) or super-diffusive (α > 1). The autocorrelation function of the velocities (ACF) determines the correlation of cell velocities at different time lags. The turning-angle distribution was defined as the occurrence of the angle between two consecutive displacement vectors, providing statistical information regarding directional movement. The MSD curve of each trajectory of DCs was collected up to τ = 60 min and fitted to the power law to calculate D and α. Only cells with R^2^ > 0.85 were used; over 74% of cells satisfied this condition. The turning angle distribution was presented depending on a time lag of 1–12 min with a bin size of 30°. 

## 3. Results

### 3.1. Environmental Mechanical Stiffness Modulates DC Morphology

**Figure 1:**
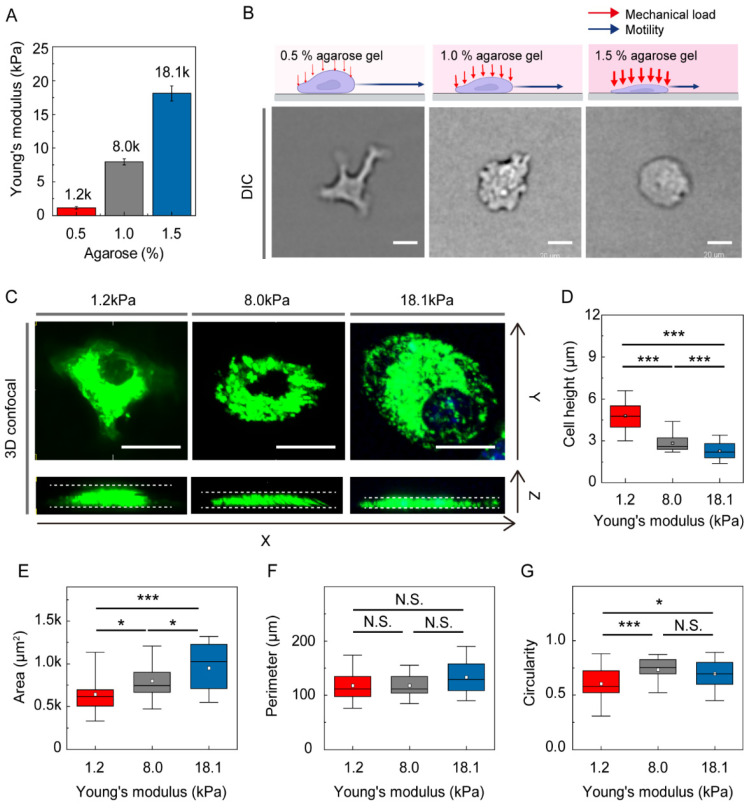
DC morphology is modulated by mechanical stress exerted by surrounding gels. (**A**) The Young’s modulus of agarose gel. Bars indicate mean value and error bar represent standard deviation (S.D.) (**B**) Schematic figure of DCs under agarose gels with different stiffness. The DIC brightfield images of DCs confined under different level of mechanical loads show distinctively different cellular morphology. Scale bar = 20 µm. (**C**) Morphological changes of DCs under compressive force were imaged by 3D confocal microscope images. Scale bar =20 µm. (**D**) Cell height, (**E**) cell area, (**F**) perimeter, and (**G**) circularity of DCs adapted under different level of mechanical loads given by the surrounding agarose gels were characterized. In figure **D**–**G**, Kruskal–Wallis/Dunn’s multiple comparisons test was used for comparisons of different sample populations; N.S.: *p* > 0.05; *: *p* < 0.05; ***: *p* < 0.001. In the box plots, the bars include 95% of the data, the box contains 75% of the data, the center bar corresponds to the median and the square point represent the mean. Data were pooled from three independent biological replicates. 1.2 kPa: *n* = 111; 8.0 kPa: *n* = 93; 18.1 kPa: *n* = 112.

To recapitulate soft confined spaces present in tissues, a gel confiner [25] was used. Three concentrations of agarose (0.5%, 1.0%, and 1.5%) were prepared to mimic soft tissues of different stiffnesses (Figure 1A). Adaptive responses of DCs depending on the mechanical load as a compressive force were clearly visible in morphology differences (Figure 1B). To observe more detailed morphologies, DiO-stained DCs were imaged using 3D confocal imaging (Figure 1C). We found that cell height was distinctively increased at low mechanical load (1.2 kPa) (Figure 1D). At higher mechanical loads with Young’s moduli of 8.0 and 18.1 kPa, DCs were more flattened, with increased surface area (Figure 1E). Interestingly, cellular perimeters remained similar regardless of the stiffness of the environment, resulting in a more spherical shape of DCs under higher mechanical loads (Figure 1F,G).

### 3.2. Stiffness of the Environment Modulates DC Motility

**Figure 2:**
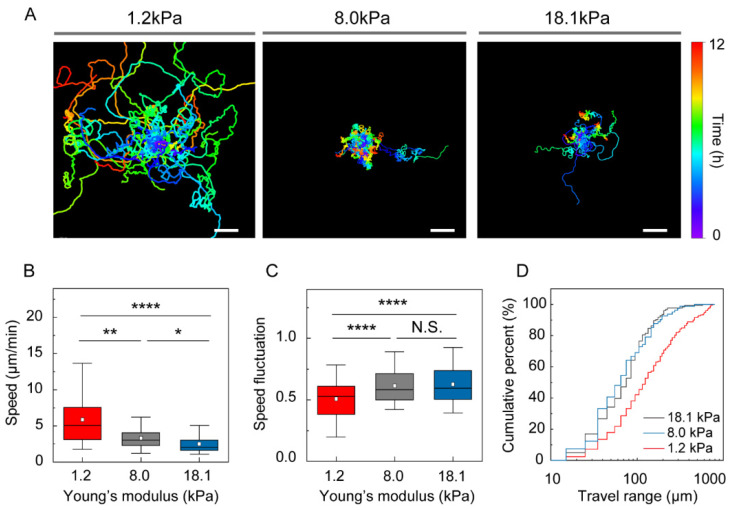
2D random motility of immature DCs under different mechanical loads. (**A**) Representative DC trajectories during the 12 h, with time-dependent color coding. The starting point of each trajectory was translated to the origin of the plot. One representative experiment of three is shown. Scale bar = 100 µm. (**B**) Mean track speed and (**C**) speed fluctuation of DCs as a function of stiffness of the surrounding gel. In the box plots, bars include 95% of the data, boxes contain 75% of the data, center bars are medians, and square points are means. Kruskal–Wallis/Dunn’s multiple comparisons test used for comparisons of different sample populations; N.S.: *p* > 0.05; *: *p* < 0.05; **: *p* < 0.01; ****: *p* < 0.0001. (**D**) Cumulative percent of the DC travel range indicating how far DCs move during the 12 h observation period. Data were pooled from three independent biological replicates; 1.2 kPa, *n* = 195; 8.0 kPa, *n* = 123; 18.1 kPa, *n* = 81.

To determine whether the intrinsic motility of immature DCs changes as a function of external mechanical load, motility was recorded at 1 min intervals for 12 h. Contrary to previous reports that DC motility is insensitive to 2D substrate stiffness [23,24], we observed a significant effect of mechanical load on motility under confinement (Figure 2A and Supplementary Videos S1–S3). DCs under a compliant gel (E = 1.2 kPa) exhibited approximately 2-fold greater mean track speeds, 6.1 ± 0.3 μm/min (mean ± S.E.) than DCs confined under stiffer agarose gels (3.5 ± 0.1 and 2.8 ± 0.1 μm/min for gels with E = 8.0 and 18.1 kPa, respectively) (Figure 2B). The speed fluctuation was lower for the DCs under compliant than stiff gels (Figure 2C). 

Interestingly, the adaptive motility response of DCs under compressive force was not linear. While a distinctively rapid navigation behavior was observed for DCs under compliant gels with weak mechanical loads (E = 1.2 kPa), motility was significantly reduced under higher compressive force (E = 8.0 kPa) but insensitive to an additional increase in mechanical load (E = 18.1 kPa).

Next, we investigated how the increased speed and low-speed fluctuation of immature DCs surrounded by compliant gel (E = 1.2 kPa) affected their function in pathogen search. As a simple measure to test how mechanical load influences DC space exploration, we measured the travel range, or the distance from the track starting point to the farthest coordinates. As shown in Figure 2D, the cumulative percentage of cells reaching up to a fixed value of travel range is lower for low mechanical loads, which indicates that more cells travel further and that DCs explore a broader area.

### 3.3. Analysis of DC Migration as Anomalous Diffusion under Mechanical Loads

**Figure 3:**
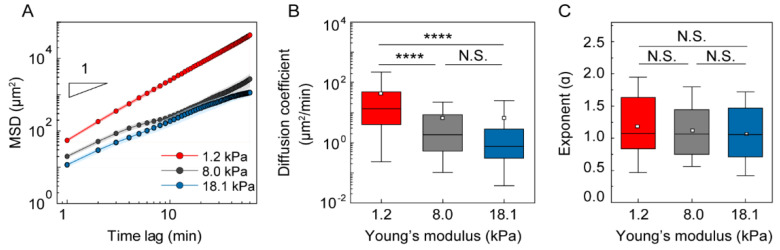
Statistical analysis of DC motility under different mechanical loads. (**A**) MSD was plotted to demonstrate the persistent random motion of DCs. Lines indicate the mean value of MSD from three independent experiments and error bars represent S.E. (**B**) Diffusion coefficient, (**C**) exponent alpha, α, pooled from three independent biological replicates. Kruskal–Wallis/Dunn’s multiple comparisons test used for comparisons of different sample populations; ****: *p* < 0.0001, N.S.: *p* > 0.05. In the box plots, the bars include 95% of the data, the box contains 75% of the data, the center bar corresponds to the median and the square point represent the mean. Data were pooled from three independent biological replicates. 1.2 kPa: *n* = 148; 8.0 kPa: *n* = 98; 18.1 kPa: *n* = 69.

MSD analysis is a useful approach for investigating intrinsic cell motility as anomalous diffusion [3,30,31,32,33]. Using 2D trajectories of the cells migrating under gels with different stiffnesses, we examined multiple statistical functions, including MSD, ACF, and turning angle distribution, using a previously reported protocol [28]. First, we obtained the average MSD in response to different mechanical loads, confirming that the diffusivity of the cells under soft gel was higher than under stiffer gels up to a time lag of 60 min (Figure 3A). The diffusion coefficient and exponent alpha were obtained by fitting the single-cell trajectory to the power-law form. As shown in Figure 3B, a significantly increased diffusion coefficient was confirmed in DCs migrating under more compliant gel with E = 1.2 kPa; D = 13.2 ± 6.2 μm^2^/min, mean ± S.E., compared with stiffer gels with higher E = 8.0 kPa (D = 1.9 ± 1.4 μm^2^/min) and E = 18.1 kPa (D = 0.8 ± 2.6 μm^2^/min). Despite the difference in diffusion coefficients, the diffusivity exponent alpha was not clearly different for different gel stiffnesses (Figure 3C).

**Figure 4:**
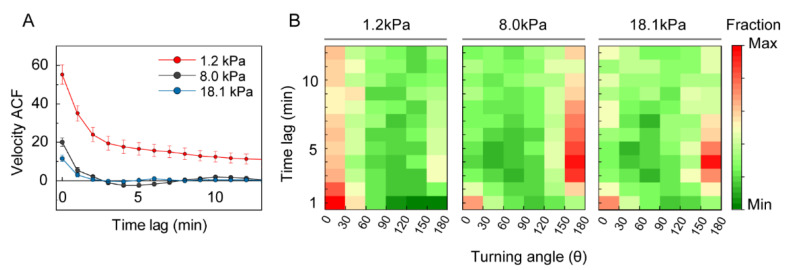
ACF and turning angle distribution of DCs migrating under different mechanical loads. (**A**) Velocity ACFs plotted to depict the velocity correlation at different time scales. Lines indicate mean ACFs from three independent experiments and error bars represent S.E. (**B**) Turning-angle distribution depending on time lag. Squares depict the fraction of the turning angle (bin size = 30°), indicated by relative color coding. Data were pooled from three independent biological replicates; 1.2 kPa, *n* = 195; 8.0 kPa, *n* = 123; 18.1 kPa, *n* = 81.

The velocity ACF is a useful parameter for quantifying the correlation of cell velocities at different time lags, indicating the timescale-dependent persistence of motility [28]. Briefly, a positive, high ACF indicates more persistent movement. If ACF is closer to 0, this indicates that the cells follow random walk motility. Negative values of ACF indicate that movement could be reversed at the corresponding time scale. The velocity ACF had a positive value for at least 15 min for the soft 1.2-kPa gel (Figure 4A). However, it was positive for only 3 min, then became negative and converged to 0 at increased time scales for the stiffer gels (8.0 and 18.1 kPa). These data indicate that the cells under lower mechanical loads move more freely in a more persistent manner than the cells under higher loads.

Motility persistence can also be measured using turning-angle distribution [28]. When motility is random, the turning-angle probability is random. If the velocity ACF is positive, the motion is persistent, resulting in a higher probability of a turning angle close to 0°. If the motility is spatially anisotropic, the turning-angle distribution has a higher value along the 0° and 180° directions. Immature DCs covered with 1.2-kPa gel had higher probabilities of turning angles below 30° (Figure 4B). In contrast, the probability of turning angles of 150–180° increased at approximately 4 min for DCs under higher mechanical loads (E > 8.0 kPa). This result suggests that the persistence time of intrinsic motility of immature DCs under high mechanical load (E > 8.0 kPa) appears to be approximately 3–4 min, but it increases up to 12 min for cells under weak mechanical load (E = 1.2 kPa).

## 4. Discussion

**Figure 5:**
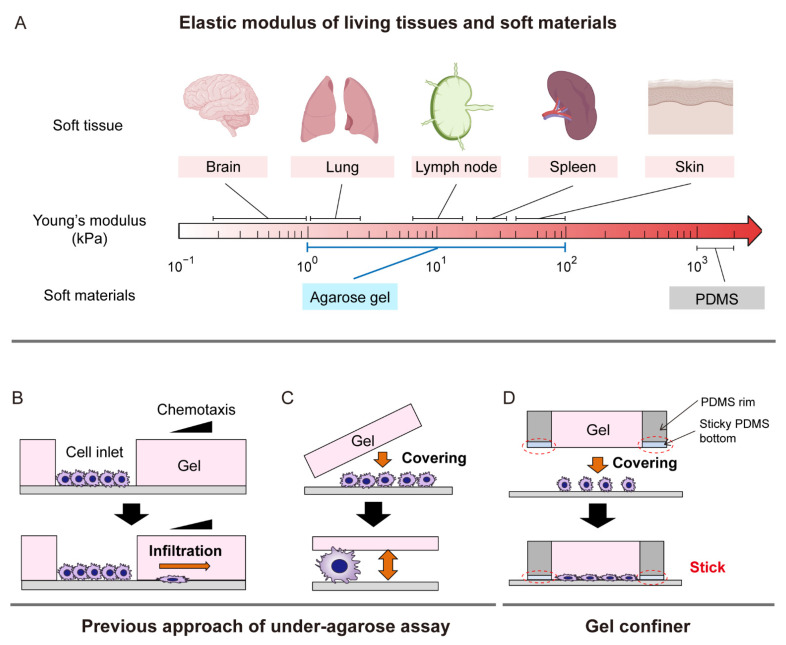
Experimental approaches to study cellular migration under confinement. (**A**) Elastic moduli of representative tissues in which DC motility has been intensively studied [21,22,35]. While DC motility has been intensively studied using PDMS microstructures, the elastic modulus of agarose gel better mimics these soft tissues. (**B**) Channel−like structures [36] and (**C**) bulk gel confinement [34] are under−agarose assay for studying amoeboid cell migration under confinement. (**D**) The gel confiner allows the handling of soft gels that better mimic confined in vivo environments [25].

DC migration in mechanically complex tissue microenvironments is a critical aspect of the immune response and consequently, disease progression. The effects of physical confinement on DC motility have long been investigated [3,10,11,12,13,14,15,16,17,18,19]. Recent studies have shown that DC motility is insensitive to the stiffness of 2D substrates [23,24]. However, DC motility on 2D substrates may not fully recapitulate DC motility under a confinement, more tissue-like geometry. Therefore, we investigated the locomotion of DCs that were completely covered by soft agarose gels with different Young’s moduli (1.2, 8.0, and 18.1 kPa). We prepared custom-designed gel confiners with elastic moduli ranging from 1 to 20 kPa by adjusting the concentration of agarose in the gel (Figure 5A). Compared to stiff PDMS microstructures with elastic moduli larger than 1 MPa, soft gels with elastic moduli of 1–2 kPa better mimic soft tissues such as brain, lung, or stroma [9]. Gels with an elastic modulus of 20 kPa were prepared to mimic stiffer tissues such as the skin, spleen, and diseased fibrotic organs.

Covering cells with a ceiling is the simplest approach to mimic confined microenvironments. The under-agarose assay has been a popular method for recapitulating leukocyte amoeboid migration in a confined space [31]. It is performed by pouring liquid agarose into a container, such as a cell-culture dish, then making two inlets in the cured gel. This is followed by placing cells in one inlet and chemo-attractants in the other to observe infiltration (Figure 5B). While this assay provides a simple approach to study chemotactic behavior, it is not ideal for random motility studies because only a small fraction of cells can penetrate the confined space under agarose. In a similar approach to the modified under-agarose assay (Figure 5C), cells are directly covered by a cured gel block, which enables measurement of intrinsic motility without cell population bias [34]. However, the handling of soft gels remains challenging. Therefore, we recently developed the gel confiner (Figure 5D) to stably cover cells with agarose gel supported by a PDMS rim structure with a sticky bottom to stably fix the gel [25]. This allows us to investigate the intrinsic motility of DCs in a well-confined, soft environment.

Interestingly, DCs under compliant, 1.2 kPa gels exhibited more rapid motility patterns than did those under higher compressive forces of 8.0 and 18.1 kPa. By analyzing motility as anomalous diffusion, we found that DCs under compliant gels produced higher diffusion coefficients, longer persistent times, and traveling across broader spaces. Under the higher mechanical loads imposed by 8.0 and 18.1 kPa gels, DC morphology became flattened and more circular, and migration became slower and less persistent with a persistence time of 3~4 min. Taken together, we found that immature DCs under complete confinement surrounded by gels with different stiffness properties exhibited mechanical load-dependent morphology and motility changes. This provides clear evidence that physical confinement is necessary to understand the role of the stiffness of the environment on the modulation of cellular motility. It also suggests that the dynamics of the cytoskeleton that control cellular morphology and motility are slow under higher compressive forces.

Several theoretical models for persistent random walks, including the levy walk and intermittent motility, have been proposed to explain the searching behavior of immature DCs [16,17,33]. In these studies, persistence and speed of inherent random motility were considered critical aspects of the search strategy. Based on our data, we hypothesize that DCs in healthy, softer tissues such as brain, lung, or stroma (<5 kPa), quickly navigate and scan a broad area. However, the DCs under stiffer gels mimic behavior in tissues such as lymph node, spleen, skin, fibroses, or tumors (>10 kPa), migrate more slowly, change direction more frequently, and scan a specific area at a longer time scale, which is hypothesized to be linked to DC function in contacting T-cells or searching for pathogens. However, further studies are required to develop models that predict cellular motility under gel confinement with different stiffness properties.

The significance of spatiotemporal heterogeneity in the adaptation of cellular motility to mechanical loads requires further investigation. A recent study found that the microscopic spatial distribution of elasticity is heterogeneous, even in the same tissue [37,38,39]. For example, brain tissue contains both soft and stiff regions measuring 2–20 μm [39]. In bone marrow, the sites of DC differentiation and those of the sinusoid (~0.1 kPa) and trabecular bone (~1 MPa) are located close to each other [20]. Even in breast cancer, elasticity heterogeneities have been reported [38]. A recent approach mimicking heterogeneous tissue elasticity and cell type-dependent motility used a cellular-scale, patterned, elastic substrate [40]. Thus, the effect of heterogeneous elasticity of the external environment on cellular migration and function requires further investigation.

We found that the DCs covered by 8.0 and 18.1 kPa gels had similar motility patterns as those under 1.2-kPa gels. With the current limited set of data, it is not clear whether there is a linear change in cellular adaptation under the mechanical load applied by the gels with elastic moduli between 1.0 and 8.0 kPa. However, it is interesting to note that the persistence time of 3–4 min that we observed for DCs under agarose gels with Young’s moduli higher than 8.0 kPa was similar to that reported for immature DCs confined in 3D collagen gel (persistence time = 2.6 ± 1.1 min) [11]. Although this will require further investigation, the present work provides a good foundation in the right range of stiffness.

There are a number of physical factors that should be considered, apart from substrate viscoelasticity, to understand cell migration in complex tissue microenvironments. Young’s modulus has generally been used as a representative property of substrate mechanical property. However, recent studies have proposed that various forms of mechanical properties could lead to unexpected results because of the complexity of the extracellular matrix (ECM) [41]. For example, stress relaxation-controlled substrates induce rapid motility in HT1080, MDAMB231, and MCF10A, which are known to show slow mesenchymal migration on soft substrates [42]. Tissue plasticity has also been suggested as a mechanical property that controls cellular motility in a cancer metastasis model [43]. To understand the motility of DCs in complex tissue microenvironments in vivo, it will be essential to determine the effects of mechanical stress tuned by not only the elastic modulus, but also by the loss modulus of the substrate or other geometrical parameters (porosity, embedded fibers, and other additives). Furthermore, unexpected synergy can arise from studying multiple physical factors independently [44]. Independent control of both geometric confinement and substrate modulus revealed that stiff and narrow confinement induces a cancer mesenchymal–amoeboid transition [45]. In another study, engineered T-cells were able to control their migration mode on a tumor-mimicking nanotextured elastic substrate [46]. Therefore, controlling elasticity alone may not be sufficient to emulate cell motility in complex tissue environments.

## 5. Conclusions

We found that the DC surveillance strategy was adopted by the surrounding mechanical load. For DCs under a low mechanical load, cellular motility is fast and persistently random, resulting in an increased travel range to scan a broader area. In contrast, cells under higher mechanical load presented with slow motility with a smaller persistent time, indicating the ability to potentially perform more thorough scanning of a small area. This distinctively different motility pattern change occurs in the elastic modulus between 1.2 and 8.0 kPa of Young’s modulus, which is a physiologically relevant range. We believe the new insight presented from this study, that is, the influence of mechanical loads on fully confined DC motility, will inspire further research to understand cellular motility in more complex tissue microenvironments, where spatiotemporal heterogeneity in terms of mechanical, geometrical, and biochemical stress exists.

## Data Availability

The data that support the findings of this study are available on request from the corresponding author.

## Supplementary Materials

The following are available online at https://www.mdpi.com/article/10.3390/cells10123362/s1, Supplementary Methods, Figure S1: Phenotypes of DC populations. Figure S2: Shear moduli of agarose gels measured by rheometry. Video S1: Dendritic cell motility under 0.5% agarose gel confinement with a Young’s modulus of 1.2 kPa. Video S2: Dendritic cell motility under 1.0% agarose gel confinement with a Young’s modulus of 8.0 kPa. Video S3: Dendritic cell motility under 1.5% agarose gel confinement with a Young’s modulus of 18.1 kPa. In all three supplementary videos, trajectory colors indicate instantaneous speeds, from 0 to over 12 μm/min.
