# Supplementary material for: Dendritic Cell Migration Is Tuned by Mechanical Stiffness of the Confining Space
Source: Cells. 2021 Nov 30;10(12):3362. doi: 10.3390/cells10123362 (PMC8699733; doi:10.3390/cells10123362)
Supplement: Supplementary file 1 [file cells-10-03362-s001.zip › cells-1460486-supplementary.pdf]

Electronic Supplementary Information

The Supplementary Information file includes:

- Supplementary Materials and Methods
- Supplementary Figure S1–S2
- Supplementary Videos S1–S3

Supplementary Materials and Methods

Preparation of bone marrow-derived DCs (BMDCs)

Tibias and femurs from BALB/c mice (8–12 weeks, female, Hyochang science, Deagu, Korea) were flushed to collect bone marrow (BM). ACK lysis buffer (Gibco, Amarillo, TX, US) was used to lyse red blood cells in the harvested BM. Bone-marrow cells were plated on tissue-culture plates (1×10^6 cells/mL) in complete medium (RPMI 1640 supplemented with 5% (*v/v*) fetal bovine serum (FBS), 1% (*v/v*) antibiotic–antimycotic solution, 1% (*v/v*) HEPES buffer, and 0.1% (*v/v*) 2-mercaptoethanol). During DC differentiation, 20 ng/mL recombinant murine granulocyte macrophage colony-stimulating factor (GM-CSF; Peprotech, Cranbury, NJ, USA) was added to the complete medium. The medium was exchanged for fresh complete medium containing GM-CSF every two days. On day 6, non-adherent and loosely adherent cells were collected by gentle pipetting, transferred to other Petri dishes, and incubated overnight. Subsequently, only floating cells were used as BMDCs.

Antibodies against the following antigens were used for the flow cytometry: MHCII I-A/I-E - FITC (clone M5/114.15.2, 1:800, Thermo Fisher Scientific, MA, US), and CD11c-APC (clone HL3, 1:200, BD Biosciences, Franklin Lakes, NJ, USA). Data were analyzed using FlowJo software (BD, Ashland, OR, USA).

Fabrication of agarose gel confiners

Mixtures of PDMS (Sylgard 184 Silicone Elastomer Kit; Dow Corning, Midland, MI, USA), with a 10:1 ratio (w/w) of prepolymer to curing agent, were used to create the main body structure. A 30:1 PDMS mixture was thinly spread on a polycarbonated sheet manually and cured overnight (65 °C) to create a sticky substrate. Subsequently, oxygen plasma treatment (80 W, 80 sccm, 1.5 min, femtoscience, Seoul, Korea) was performed on both the cured thin, sticky PDMS sheet and bottom side of the PDMS main body to bond them. The PDMS structure was placed on a Petri dish before the gel was cast. Low-melting agarose was prepared at 0.5%, 1%, and 1.5% (*w/v*). In detail, twice the final concentration of low-melting agarose was dissolved in phenol red-free HBSS buffer (Gibco) by gentle magnetic stirring at 90 °C for 20 min. The melted agarose solution was slowly cooled to 40 °C, and an equal volume of warm (37 °C) 2× conditioned medium (RPMI 1640, 10% [v/v] FBS, 2% [v/v] HEPES buffer, 2% [*v/v*] antibiotic-antimycotic solution, 0.2% [*v/v*] 2-mercaptoethanol) was added to give a final agarose gel concentration of 1×, which was cast onto the PDMS structure. The gel integrated with the PDMS structure, was cured for 20 min at room temperature, then was kept in medium and incubated overnight.

Morphology

DC morphology was examined by DIC brightfield imaging with a 10× dry objective lens. Detailed DC morphology under gel confinement was characterized using laser scanning confocal imaging (A1R, Nikon, Melville, NY, US). The DC suspension stained with DiO (Invitrogen, Waltham, MA, US) was seeded on a 20 μg/mL FN-coated glass substrate and

incubated for 20 min before being covered with a gel confiner. Fluorescence Z-stacks were acquired using a 100× Plan Apo lens with a 0.5 μm *z*-interval. Cell height was measured manually. Z max intensity projected images were semi-automatically segmented as single cells to analyze 2D morphological characteristics such as cell area, perimeter, and circularity. Circularity was calculated using the equation:

$$Circularity = \frac{4 \times \pi \times Area}{perimeter^2}$$

More than 10 cells were measured at each of five positions from three independent samples. Morphological analyses were performed using NIS Elements (Nikon, Melville, NY, US).

DC motility analysis

Background-subtracted bright-field images were used to track non-labeled DC motility as previously described [1]. Cell tracks were automatically detected using the Imaris (Bitplane, Zürich, Switzerland) "Spots" function. Cells were identified using intensity thresholding and object diameter estimated to be 20 μm. Tracking errors such as disconnected tracks were manually corrected. Previous studies provided reasonable filtering criteria to remove artifacts, noise, and dead or dying cells from raw data when dealing with trajectory analysis [2]. In the current work, three similar filtering criteria were applied: mean track speed (≥1.5 μm/min), track duration (≥60 min), and maximal distance from starting position (≥20 μm).

Supplementary figures

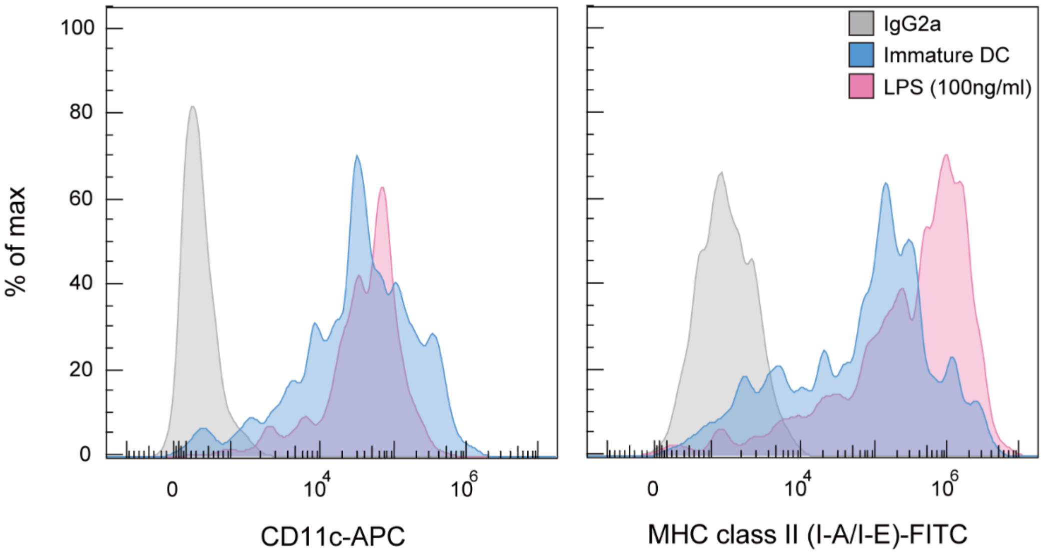

Figure S1. Phenotypes of DC populations. Expression levels of CD11c and MHC class II (I-A/I-E) molecules measured by flow cytometry. To verify characteristics of harvested DCs, both immature and LPS-treated DCs were investigated in every independent experiment. One representative experiment of three is shown.

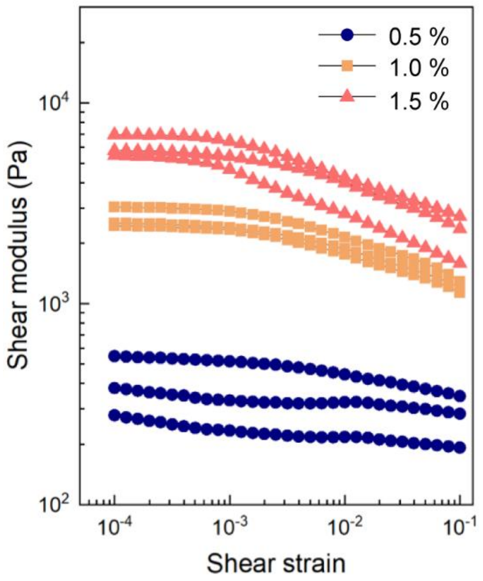

Figure S2. Shear moduli of agarose gels measured by rheometry. Range of shear strain between 10^{-4} and 10^{-3} were selected to calculate elastic Young's moduli. Three independent experiments were performed.

References

1. Choi, Y.; Sunkara, V.; Lee, Y.; Cho, Y.-K. Exhausted mature dendritic cells exhibit a slower and less persistent random motility but retain chemotaxis against CCL19. *Biorxiv* **2021**, doi:10.1101/2021.10.11.463881.
2. Stankevicins, L.; Ecker, N.; Terriac, E.; Maiuri, P.; Schoppmeyer, R.; Vargas, P.; Lennon-Dumenil, A.M.; Piel, M.; Qu, B.; Hoth, M.; et al. Deterministic actin waves as generators of cell polarization cues. *Proc. Natl. Acad. Sci. U. S. A.* **2020**, *117*, 826–835, doi:10.1073/pnas.1907845117.

Supporting Video S1: Dendritic cell motility under 0.5% agarose gel confinement with a Young's modulus of 1.2 kPa. Trajectory colors indicate instantaneous speed per 1 min.

Supporting Video S2: Dendritic cell motility under 1.0% agarose gel confinement with a Young's modulus of 8.0 kPa. Trajectory colors indicate instantaneous speed per 1 min.

Supporting Video S3: Dendritic cell motility under 1.5% agarose gel confinement with a Young's modulus of 18.1 kPa.

In all three supplementary videos, trajectory colors indicate instantaneous speeds, from 0 to over 12 μm/min.
